# Obesity and Diabetes in the Winnebago Tribe of Nebraska: From Community Engagement to Action, 2014–2019

**DOI:** 10.5888/pcd16.190181

**Published:** 2019-08-08

**Authors:** Larry Alonso, Lorelei Decora, Ursula E. Bauer

**Affiliations:** 1 Tribal, Regional, and Territorial Support Team, Program Implementation Branch, Division of Diabetes Translation, Centers for Disease Control and Prevention, Atlanta, Georgia; 2 Winnebago Public Health Department, Winnebago, Nebraska; 3 Office of the Deputy Director for Noninfectious Disease, Centers for Disease Control and Prevention, Atlanta, Georgia

## Abstract

The Winnebago Tribe of Nebraska implemented interventions to promote the health of their people, focusing on community-selected and culturally adapted policies, systems, and environmental (PSE) improvements to reduce the prevalence of obesity and type 2 diabetes. The interventions were implemented as part of the Centers for Disease Control and Prevention’s (CDC’s) 2014–2019 Good Health and Wellness in Indian Country program. The Winnebago Tribe used CDC’s CHANGE community health assessment tool to prioritize and direct their interventions. They integrated findings from a community health assessment tool with observations from tribal working groups and implemented 6 new evidence-based PSE interventions. Their successful approaches — selected by the Winnebago community, culturally relevant, and driven by scientific assessment —demonstrate the value of flexibility in CDC grant programs.

**Summary**

**What is already known on this topic?**

Policies, systems, and environmental (PSE) changes are evidence-based interventions known to lead to lasting public health improvements.

**What is added by this report?**

PSE improvements can be culturally relevant, can be led by tribal health leadership working respectfully with community members and across sectors to meet expressed community needs, and can solve practical problems that constitute everyday barriers to health and healthy behaviors.

**What are the implications for public health practice?**

Public health practitioners may consider flexible, locally led approaches to improving health outcomes that build on the expressed needs of the community.

## Introduction

Of the 9.4% of the US population who had diabetes in 2015, most (90%–95%) had type 2 diabetes (1). The prevalence of diabetes in the United States is highest among American Indian/Alaska Native adults: in 2015, prevalence was 14.9% among men and 15.3% among women aged 18 years or older (1). The age-adjusted prevalence of diagnosed diabetes among American Indian/Alaska Native adults varies by region, from a low of 6.0% in Alaska to a high of 22.2% in some areas of the Southwest (1). Obesity is a major risk factor for type 2 diabetes and is itself associated with serious health risks (2). According to the National Health and Nutrition Examination Survey, during 2011–2014, 17.2% of people aged 2 to 19 met the definition of obesity (body mass index [BMI] >95th percentile), including 20.5% of adolescents and young adults aged 12 to 19. During that study period, the prevalence of obesity among adults was 36.0% (3). With obesity and diabetes affecting the health of so many Americans and disproportionately affecting American Indians and Alaska Natives, finding culturally relevant ways to implement effective interventions is essential (4). The Winnebago Tribe of Nebraska sought to do just that.

## Background

The Winnebago Tribe of Nebraska reservation is home to 2,694 residents and located in rural northeastern Nebraska and northwestern Iowa, 20 miles south of Sioux City, Iowa, and 80 miles north of Omaha, Nebraska, on both sides of the Missouri River. Most residents live on the western side in or near the village of Winnebago. Almost 40% of the population is younger than 20 years, and 18% are aged 20 to 34 (5). During the 2013–2014 school year, 429 children attending a school in the Winnebago public school district or the St. Augustine Indian Mission School in kindergarten through 12th grade were screened for risk factors associated with type 2 diabetes, including BMI. Of these children, 232 (54.1%) had a BMI in the 85th percentile or higher (indicating overweight or obesity). This information drove the priorities of the Winnebago Tribe’s Good Health and Wellness in Indian Country (GHWIC) grant from the Centers for Disease Control and Prevention, a 5-year cooperative agreement launched in 2014 (6). The tribe conducted a community health assessment, convened a cross-sector work group, and implemented a community action plan consisting of community-chosen and culturally adapted interventions to improve policies, systems, and environments (PSE) to promote health, with a focus on obesity and diabetes across the age span. The GHWIC program encouraged several risk-reduction strategies, such as reducing commercial tobacco use, increasing healthful nutrition and regular physical activity, improving health literacy, and deploying the National Diabetes Prevention Program (7) and the Million Hearts hypertension control strategy (8).

## The Good Health and Wellness in Indian Country Program

The Winnebago Tribe used CDC’s Community Health Assessment aNd Group Evaluation (CHANGE) tool to conduct a survey to understand the community’s needs and priorities (9). The tool consists of 5 sectors: community-at-large, community institution/organization, health care, school, and worksite. The Winnebago Tribe convened a cross-sector work group to review CHANGE findings, identify assets and needs in each of the 5 sectors, and prioritize strategies and activities to achieve outcomes of importance to the community. For example, survey results for the community/organization sector showed that 42.7% (38 of 89) of children screened in Head Start had overweight or obesity. This led the Winnebago Tribe to select infant nutrition and parental education as a priority. The community health assessment also identified challenges, such as insufficient housing for community members, inconsistent tobacco-free policies in tribal buildings, absence of nutrition standards across tribal nutrition and food programs, and absence of a tribal employee wellness policy. The cross-sector working group reviewed 15 sets of data collected with the CHANGE tool and prioritized the following core GHWIC intervention areas: physical activity, nutrition, tobacco, chronic disease management, and leadership, with a focus on PSE strategies for implementation. To ensure continued community buy-in, key community members continued to participate in the cross-sector working group and engage in each new step of implementing the community action plan. This community buy-in helped ensure that the plan met needs of the community. Progress toward program implementation and completion is documented through an annual performance report and telephone calls between CDC project officers and program staff members.

## Outcomes

The Winnebago Tribe of Nebraska achieved outcomes across the PSE strategies they implemented.

**Public health nursing referral policy**. This policy addresses a key CHANGE finding: community members recently discharged from area hospitals are lost to follow-up, resulting in chronic disease exacerbation. The Winnebago Tribal Health Director approved the Winnebago Public Health Nursing Referral Policy for Hospital Discharge in April 2017. This policy provides instructions to medical facilities in the Sioux City area for Winnebago residents who are discharged. The policy asks “all discharging facilities to forward their discharge referrals and instructions specific to the diagnosis or procedure, labs, summaries, medication prescriptions, and durable equipment prescriptions to the Winnebago IHS [Indian Health Service] Social Worker and/or Nurse Case Manager.” Winnebago patients are then contacted by the Winnebago IHS social worker and public health nursing department for follow-up and post-hospital visits to prevent complications and hospital readmission.

As a result of this policy, the tribe reported reduced hospital readmissions, improved continuity of care and follow-up with Winnebago health care resources, and improved health education for the discharged patient and family. Relevant to this policy change is that on July 1, 2018, the Winnebago Tribe of Nebraska under Title V of the Indian Self Determination Act (Self-Governance) assumed the governance of the Winnebago Indian Health Service Hospital. The Hospital was renamed the 12 Clans Unity Hospital on that date. This administrative change will help ensure policy sustainability by removing potential interagency barriers between policy design and implementation.

**Allow community health representatives (CHRs) access to IHS electronic health records.** CHRs visiting patients in homes and other nontraditional health care settings are often the first people to spot issues amenable to outpatient management. However, CHRs lack official channels for communicating critical actions to promote health and prevent illness and injury. Furthermore, CHRs were not authorized to enter clinical findings and measures into IHS electronic health records (the Resource and Patient Management System), limiting their ability to share information with health care providers. The new Winnebago policy specifies procedures for CHRs to enter information into the patient’s IHS electronic health record.

This policy will improve case management. It will also create early intervention opportunities for patients referred to CHRs by health care providers working for the Winnebago Public Health Department or IHS and improve chronic disease management, patient follow-up, communication between CHRs and health care providers, and referrals to community resources. The policy, officially adopted in September 2017, was implemented in beta format in February 2019.

**Tribal sugar-sweetened beverage (SSB) tax initiative.** Recognizing the contribution of sugary drinks to obesity and diabetes and the interest in addressing the availability of SSBs identified in the community health assessment, and after reviewing data and information gathered through the GHWIC program, the Winnebago Tribe proposed taxing SSBs to the Tribal Council’s vice chairman in October 2018. Such a tax would reduce consumption and provide revenue for public health interventions. The proposal applies to SSBs sold by retailers within the exterior boundaries of the Winnebago Indian Reservation at a rate of $0.02 per fluid ounce. Milks, infant formula, and beverages for medical use are exempt. The tribe proposes to use resources collected under the new law to fund nutrition and physical activity interventions.

If the policy is adopted and implemented, the tribe anticipates outcomes will be consistent with those of localities, states, other countries and other tribes that have implemented similar policies. Development of this policy has been slow and thoughtful because of the support needed from retailers and the details of tracking and collecting taxes. The Winnebago Tribal Council continues to consider this proposal and the potential health and economic impact of such a tax.

**Infant and childhood obesity prevention health education program.** To prevent overweight and obesity in infancy and early childhood, the Winnebago Tribe is using education and prevention initiatives aimed at parents and grandparents who are direct caretakers. The Winnebago Public Health Department implemented complementary health education efforts addressing each stage of life, with mutually reinforcing messages across all tribal programs. The Winnebago Tribe implemented age-appropriate health education for child caregivers in 7 settings:

- the Educare parent education room (Educare is a model for early childhood education [10]);
- office visits to the tribe’s registered dietitian;
- Educare Head Start, Winnebago Public School K–2nd , and St. Augustine K–2nd receiving the *Eagle Book* series (stories for young children that highlight the wisdom of traditional ways of health [11]);
- middle and high school classes at Winnebago Public School and middle school classes at St. Augustine Indian Mission School using the *Youth Staying Healthy* curriculum (an IHS curriculum used by health care professionals to provide diabetes prevention education to children aged 8–12 and their family members in one-on-one or group settings [12]);
- the Senior Center Lunch and Learn program;
- the Winnebago Indian Health Service/Twelve Clans Unity Hospital, integrated into health fairs, prenatal nutrition, and physical activity education; and
- the Native Lifestyle Balance Program at the Little Priest Tribal College.

Outcomes of these interventions are being evaluated as part of the Good Health and Wellness in Indian Country project, concluding in September 2019. Preliminary qualitative and quantitative process outcomes suggest improvements in knowledge and attitudes among participants.

**Winnebago Tribe healthy foods and beverages policy.** The community health assessment revealed that the ready availability of poor food choices, along with SSB availability, likely contributes to high levels of obesity in the community. In 2016, the Winnebago Public Health Department adopted and implemented a healthy food and beverages policy. Initial discussions of the cross-sector working group showed support for a policy prohibiting SSBs and low-nutrition (“junk”) foods from being served at Winnebago Tribe of Nebraska workplaces and events, fundamentally changing the quality of foods and beverages offered.

The shared understanding and resolve of the community to address nutritional drivers of chronic disease is expected to lead to changes in attitudes and behaviors and to improve health outcomes related to nutrition, obesity, and chronic diseases over the long term.

**CDC-recognized type 2 Diabetes Prevention Program.** The Winnebago Tribe was one of the original participating sites in the type 2 diabetes prevention demonstration project, Special Diabetes Program for Indians. The study results, published in 2013, demonstrated the feasibility of implementing a lifestyle intervention in diverse American Indian and Alaska Native communities to prevent or delay onset of type 2 diabetes in adults at high risk (13). As part of GHWIC, the Winnebago Tribe implemented the Native Lifestyle Balance curriculum and pursued CDC recognition as part of CDC’s National Diabetes Prevention Program. With support and technical assistance from the Great Plains Tribal Chairmen’s Health Board, Winnebago trained 3 lifestyle coaches and received pending recognition from CDC’s Diabetes Prevention Recognition Program. With support from the American Association of Diabetes Educators, 2 reimbursement specialists (staff members trained to bill for reimbursable type 2 diabetes prevention services) were trained, increasing the potential for long-term sustainability of this proven type 2 diabetes prevention program. Effective implementation of the program over the long term has the potential to change the trajectory of type 2 diabetes among the Winnebago.

**Pedestrian safety and built environment.** In collaboration with the Winnebago Public School, the Village of Winnebago, and the Winnebago Tribe, the Nebraska Department of Roads installed rectangular flashing beacons signaling pedestrian crossings at key downtown locations to facilitate safe street crossing, including crossings to health promotion facilities and services. Additionally, in 2018, a traffic circle was funded for construction by the Nebraska Department of Roads after findings of the community health assessment prompted telephone calls to state officials about speed reduction measures on State Highway 77, which bisects the reservation. Construction of the traffic circle is currently underway, with completion anticipated in fall 2019.

These measures have the potential to increase safe pedestrian activity in this high-traffic area of the reservation and to build additional demand for further walkability improvements.

## Implications for Public Health Practice

The Winnebago Tribe of Nebraska nurtured opportunities provided by the GHWIC program to create PSE improvements addressing obstacles to good health in response to information provided by community leaders and residents. By adapting GHWIC resources and tools to local processes and approaches and keeping community needs front and center, the Tribe introduced, implemented, or completed effective PSE improvements that, over time, will lead to improved health outcomes. The process undertaken by the Winnebago Tribe was one of rich, ongoing community engagement, shaping CDC’s tools to be relevant to the tribe’s health goals while helping tribal members take action to advance those goals. The result is an array of improvements driven by the expressed needs of the tribe, accomplished through mutually respectful collaboration and generous support from the community.
